# The All4Children project to assess the initial implementation of the Integrated Model of Family Foster Care in Portugal: A description of the study protocol

**DOI:** 10.1371/journal.pone.0304244

**Published:** 2024-05-24

**Authors:** Joana Baptista, Helena Grangeia, Mariana Negrão, Cláudia Camilo, Sandra Ornelas, Sandra Nogueira, Isabel Pastor, Ana Gaspar, Isabel Soares, Stephanie Alves

**Affiliations:** 1 Instituto Universitário de Lisboa (Iscte-IUL), CIS-Iscte, Lisbon, Portugal; 2 ProChild CoLAB, Guimarães, Portugal; 3 Research Centre for Justice and Governance (JusGov), Braga, Portugal; 4 CEDH, Portuguese Catholic University, Porto, Portugal; 5 Santa Casa da Misericórdia de Lisboa, Lisbon, Portugal; 6 School of Psychology, University of Minho, Braga, Portugal; 7 HEI‐Lab: Digital Human‐Environment Interaction Labs, Lusófona University, Lisbon, Portugal; Public Library of Science, UNITED STATES

## Abstract

**Background:**

The All4Children project addresses the urgent need to transition from institutionalization to family-based care for out-of-home children in Portugal. Despite evidence highlighting the detrimental effects of institutionalization, only a small percentage of children (less than 4%) are currently placed in family foster care in the country. In response to European directives for deinstitutionalization, Portuguese legislation now prioritizes non-kinship family foster care as the preferred alternative for young children in need of care. To facilitate this transition, the Integrated Model of Family Foster Care (MIAF) was developed, offering a comprehensive framework covering the entire spectrum of family foster care.

**Objective:**

This research aims to investigate the initial implementation stage of the MIAF to promote high-quality family foster care in Portugal.

**Method:**

The study will conduct a mixed-method and longitudinal research project in family foster care agencies across different regions of Portugal, focusing on evaluating the implementation and outcomes of the MIAF model using a multi-informant and multi-method approach. The participants will include caseworkers, children aged 0–9 years entering foster care, and their respective foster families enrolled in the MIAF program. Process evaluation will assess fidelity, feasibility, appropriateness, and acceptability of MIAF modules, while outcome evaluation will examine child safety, stability, well-being, as well as foster family well-being and quality of relational care.

**Outcomes:**

The insights gained from this research initiative will serve as a foundation for the ongoing enhancement of MIAF. Consequently, this project has the capacity to advance evidence-based child welfare practices by refining processes and strategies to better serve vulnerable children and youth.

**Conclusion:**

Facilitated by a multidisciplinary team, this project will contribute to advancing research in the field, enhancing practice, and informing policy during a pivotal stage of deinstitutionalization in Portugal.

## Introduction

Every child has the fundamental right to be raised in a responsive family environment that fosters their optimal development. Unfortunately, around 8 million children worldwide currently reside in institutional care [1]. Extensive research documenting the harmful effects of institutionalisation across various domains of child functioning, particularly on infants and young children, is well-established [2]. Equally well documented is the capacity for recovery following family foster care placement [3]. However, in Portugal in 2022, only 3.6% of all out-of-home children were placed in family foster care [4]. In alignment with the European calls-to-action for deinstitutionalization [5], the Portuguese law (e.g., Decree-Law 142/2015) has recently underscored non-kinship family foster care as the preferred alternative care measure for children up to the age of 6. As a result, the country recently implemented processes for recruiting, training, selecting foster families, and placement of children in these settings.

The transition to a family foster care system presents challenges, requiring highly specialised child welfare services to address the unique needs of families and children [6]. Notably, the pitfalls in child welfare reforms are, in part, attributed to rapid changes that rely on unprepared staff and evidence-practice gaps [7]. Child welfare practice models, conceptual maps rooted in values, can contribute to addressing these challenges. These frameworks help specify and operationalize caseworkers’ skills and practices through different stages of the child welfare continuum [8, 9]. As such, child welfare practice models have been found to enhance consistency in practice [10], facilitate the achievement of case goals and family engagement [11], and improve children’s outcomes when implemented with fidelity [12].

In Portugal, the Integrated Model of Family Foster Care (MIAF) has recently emerged as a pioneering child welfare practice model to respond to the demands of a paradigm shift to a family foster care system. MIAF is designed to encompass the complete continuum of family foster care, focusing on aspects from the recruitment, training, assessment, and selection of foster families to the placement of children in foster care and their subsequent transition to a permanent solution, such as family reunification or adoption. Aligned with national laws, MIAF incorporates fundamental principles and intervention guidelines for each phase of family foster care. With multiple teams in the country starting to implement this model, it aligns with principles emphasized in implementation science frameworks [e.g., 13]. These frameworks advocate for a stage-based approach, prioritizing an initial focus on implementation process evaluations (e.g., fidelity) and child and family outcomes rooted in a well-defined theory of change before progressing to more advanced stages, including effectiveness testing and full-scale implementation [8, 13]. This methodical and comprehensive approach allows for crucial adjustments to be made to child welfare practice models before scaling-up. The focus of this research is to investigate MIAF as a novel child welfare practice model aimed at promoting high-quality foster family care in Portugal.

From a process evaluation perspective, spanning multiple levels, various factors can influence the successful implementation of a new model, such as MIAF [14]. These factors may relate to the provider (e.g., knowledge and relational skills), the innovation itself (e.g., flexibility), the client (e.g., outcomes), the organization (e.g., leadership), and the process (e.g., fidelity feedback). Identified factors can potentially act as barriers, limiting adherence to the new child welfare practice model [15, 16]. In the Portuguese context, the urgency to identify such factors is compounded by the complex, often crisis-oriented dynamics that child welfare workers must navigate. Moreover, the prevailing lack of a family foster care culture in the country [17], coupled with stakeholders’ negative perceptions of family foster care [18], underscores the significance of mapping these multi-level factors for informed decision-making in the ongoing implementation of MIAF.

Despite the significant implications for child safety, stability, and overall well-being, many studies on child welfare practice models have neglected comprehensive outcome evaluations, leaving unanswered questions regarding their effectiveness in empowering families. Recent evidence suggests that foster parents may experience distress [19] and decreased responsiveness [20] during the crucial first year of placement, a period intricately linked to child outcomes such as behavior problems [21], attachment disorders [22], and placement instability [23]. Research also underscores the importance of examining the dynamic interplay between child and family factors [24], highlighting the significant impact of both child-related factors (e.g., behavior problems) and family-related variables (e.g., stress, mental health) at the time of placement on caregiving quality. This, in turn, may adversely affect child outcomes [19, 20, 21]. Yet, the extent to which child welfare practice models serve as buffers, mitigating the impact of these early risk factors and safeguarding children, remains inadequately explored.

### Objectives of the research study

This research, guided by the Getting To Outcomes framework [8], aims to investigate the initial implementation stage of the MIAF, a child welfare practice model recently developed to address the current national priority in child welfare policies in Portugal. The goal of the MIAF is to promote high-quality family foster care practice standards and support families and children in achieving safety, stability, and well-being outcomes. Therefore, this study seeks to conduct a mixed-method process and outcomes evaluation. In terms of process evaluation, our objectives are to (1) determine whether the MIAF is being implemented as planned (i.e., with fidelity) and assess its feasibility, appropriateness, and acceptability, and (2) identify enablers and barriers to MIAF implementation. For outcome evaluation, our objectives are to (3) describe the developmental trajectories of foster care children enrolled in the MIAF, assess their safety and stability, and (4) examine the trajectories of foster parents’ well-being and the quality of relational care. Additionally, we aim to (5) evaluate whether the MIAF mitigates the potential negative impact of early risk factors at the child (e.g., behavior problems) and family (e.g., stress, psychological symptoms) levels, measured at the time of placement, on subsequent outcomes assessed up to 12 months post-placement.

## Methods

### The Integrated Model of Family Foster Care (MIAF)

The Integrated Model of Family Foster Care (MIAF) is a comprehensive framework designed to address the transition to a family foster care system in Portugal, ensuring high-quality services to meet children’s needs and support both foster and birth families. Developed by ProChild CoLAB and Santa Casa da Misericórdia de Lisboa, MIAF relies on an iterative process between researchers’ and child welfare practitioners’ expertise, fostering a collaborative approach. MIAF aims to enhance the professional and institutional practices of family foster care by promoting a common language and shared thinking, evidence-based practices, and the development of technical and relational competencies. These efforts are expected to positively impact children’s well-being and safety, as well as the (co)parenting skills of foster and birth families, and the relationships between the child, foster family, birth family, and caseworkers. MIAF incorporates a comprehensive training program for caseworkers, comprising initial training sessions, ongoing supervision, and continuous training throughout the implementation of the model. This approach ensures that caseworkers are equipped with the necessary knowledge and skills to effectively implement MIAF and provide high-quality support to foster families and children in care.

MIAF adopts a child-centered and trauma-sensitive approach [25], aiming to provide a secure base for children in foster care and promote adaptive developmental trajectories and relational permanency [26]. This focus on stable relationships contributes to a strong sense of belonging, identity, and life continuity for foster children. MIAF encompasses key processes across the entire spectrum of family foster care, each tailored to achieve specific objectives: (a) recruitment, training, assessment, and selection of foster families, designed to cultivate high-quality foster parenting by identifying, training, and assessing suitable candidates to become foster families; (b) matching children with foster families—this process guides decision-making to ensure the best match between a child in need and a suitable foster family, taking into account the child’s unique needs and circumstances; and (c) foster care placement and transition to a permanent placement—this process focuses on facilitating high-quality coparenting care, e.g. by engaging both foster and birth or adoptive families in shared caregiving responsibilities. The goal is to ensure a smooth transition for the child from foster care to a permanent placement, promoting stability and continuity of care.

### Overview of the research design

The research project employs a multi-method approach, incorporating both qualitative and quantitative data collection methods, along with a multi-informant approach. Process evaluation data for the MIAF key processes will be collected at two points: pre-placement (M1) and post-placement (M2). Outcome evaluation data will be collected longitudinally over the first 12 months of the child’s placement in foster care. The study will recruit 25 caseworkers engaged in implementing the MIAF from four child welfare agencies situated in the north and south of Portugal. Additionally, 100 children aged 0–9 years entering foster care and their respective foster families receiving the MIAF will be included in the study. It is expected that this sample will cover nearly the entire population served by the four foster care agencies participating in the study. The recruitment period for participants will extend from March 2024 until December 2025.

### Process evaluation

Informed by Proctor et al.’s [27] taxonomy on implementation outcomes, the process evaluation will utilize both qualitative and quantitative methods to assess the key processes of the MIAF. These include implementation fidelity, which measures how closely the MIAF was adhered to as per the original protocol or the intentions of the developers. Feasibility will also be evaluated, gauging the extent to which the MIAF can be effectively implemented within the targeted agencies or settings. Additionally, acceptability will be assessed, focusing on stakeholders’ perceptions of the MIAF’s satisfaction and suitability. Furthermore, the process evaluation will identify enablers and barriers influencing the implementation of each key process within the MIAF. The participants will include caseworkers, foster families, and children in care.

#### Process evaluation—Quantitative methods

Informed by previously validated measures of implementation outcomes [28], a set of questionnaires were developed specifically for this project to assess fidelity, feasibility, and acceptance of MIAF implementation. Both caseworkers and foster families will complete these questionnaires online at different time points, including pre-placement (M1) and post-placement (M2). Regarding M1, data will be collected (a) after informative sessions for awareness raising and foster family recruitment, (b) after pre-service training, and (c) after the assessment and selection of foster families. For M2, questionnaires will be completed (d) one month following the child’s placement to assess child-foster family matching and the transition to family foster care processes, (e) at six months of placement to evaluate support during family foster care, and (d) one month following the child’s transition to a permanent solution. The number of items per questionnaire, rated on either a 3-point or 5-point scale, varies depending on the MIAF key process and informant (i.e., caseworker or foster family).

#### Process evaluation—Qualitative methods

Additionally, focus groups with caseworkers will be conducted at two stages of the research project to explore the enablers and barriers to MIAF implementation. During the first year of MIAF implementation, the focus will be on pre-placement processes (M1), while in the second year, discussions will encompass experiences related to matching, interventions during placement, and transitions in foster family care (M2). At each stage, at least three focus groups will be conducted for each participant group, with each group comprising fewer than 10 participants. The sessions will begin with an introduction to the study objectives and the participants’ role. Subsequently, informed by the framework proposed by Akin et al. [15], more in-depth questions will be asked regarding the enablers and barriers influencing MIAF implementation, including process factors, provider factors, innovation factors, client factors, organizational factors, and structural factors. All focus group discussions will be recorded, transcribed verbatim, and subjected to qualitative analysis.

Furthermore, specifically designed short semi-structured interviews will be conducted with children aged over five years. Each child will participate in an interview six months after their placement. Children will be presented with a series of open-ended questions, asked in a flexible order depending on their responses, to explore their experiences and feelings regarding foster care. The interviews will also include a warm-up drawing activity. All interviews will be audio-recorded and transcribed verbatim to facilitate qualitative data analysis.

### Outcome evaluation

The outcome evaluation will utilize a longitudinal design, gathering qualitative and quantitative data from caseworkers, foster families and children involved in the MIAF program over the initial 12 months of foster care placement. Follow-up assessments with children and their foster families are planned at 1-month (T0), serving as baseline for family and child outcomes, and subsequently at 3 (T1), 6 (T2), 9 (T3), and 12 (T4) months post-placement. Through a combination of reported and observational measures, the assessment protocol will address child development and well-being, child safety and stability, foster family well-being and the quality of relational care. Report measures will primarily be collected online, while interviews, observational measures and tasks with the child will be collected through home visits to each foster family (Table 1, for a summary of the measures and moments of data collection regarding process and outcome evaluation).

**Table 1:** Assessment protocol and assessment moments regarding outcome evaluation.

				Assessment Moments				
Variable	Informant		Method	T0	T1	T2	T3	T4
**Child Outcomes **								
Mental Development	Child		In-person	×		×		
Physical growth	Foster family		In-person	×		×		
Emotional/behavior problems	Foster family		Online	×	×	×	×	×
Well-being	Foster family		Online	×	×	×	×	×
Competence	Foster family		Online	×	×	×	×	×
Attachment disordered behaviors	Foster family		In-person			×		×
Safety and permanency	Caseworkers		Online		×	×	×	×
**Family Outcomes **								
Psychological wellbeing	Foster family		Online	×		×		×
Parental stress	Foster family		Online	×		×		×
Self-efficacy in foster parenting	Foster family		Online	×		×		×
Satisfaction in foster parenting	Foster family		Online	×		×		×
Caregiver sensitive responsiveness	Caregiver-child interaction		In-person	×		×		×
Acceptance, commitment, awareness	Foster family		In-person			×		×
Retention of the foster family	Caseworkers		Online	When (and if) a child leaves care				

Note. T0 (1 month of placement), T1 (3 months of placement), T2 (6 months of placement), T3 (9 months of placement), T4 (12 months of placement).

#### Child development and well-being

For assessing mental development, trained examiners will administer the Griffiths Mental Development Scales (0–2 and 2–8) [29]. These scales encompass six subscales: locomotor, personal and social, language, eye and hand coordination, performance, and practical reasoning (the last one only for children above 2 years). Scoring is based on reference standards that determine a functional age according to the accumulated score for each subscale. Additionally, a general quotient is obtained by averaging scores across the five subscales. For older children, cognitive ability will be evaluated using the Information and Block Design subtests of the Wechsler Preschool and Primary Scales of Intelligence (WPPSI) [30]. These subtests will provide an estimated child IQ score, following the guidelines recommended by Sattler [31]. *Physical growth* data, including height, weight, and head circumference, will be collected from the children’s medical records. Raw data will be converted into percentiles for analysis. Regarding *child emotional and behavioral problems*, foster family will be asked to complete either the Baby Pediatric Symptom Checklist for infants up to 18 months (12 items) [32], the Preschooler Pediatric Symptom Checklist for children aged 18 to 60 months (19 items) [33], or the Pediatric Symptom Checklist for school-aged children (17 items) [34], depending on the child’s age. These questionnaires are designed to assess emotional and behavioral problems in children and include questions about various types of behavior. Responses are rated on a three-point scale ranging from 0 (Not at all) to 2 (Very much), tailored to recognize behaviors typical for children in the respective age ranges. Foster families will also complete the Portuguese versions of the Secure Based Models checklists [25], which assess a child’s developmental strengths and difficulties, as an index of *well-being*. These checklists have different versions tailored to specific age ranges (i.e., 0–18 months, 19 months-4 years, 5–10 years). Items on the checklists are rated on a three-point scale: yes, sometimes, no. Additionally, caregivers are provided with open-ended questions to add any additional comments they may have. Child flourishing will also be measured using very short questionnaires consisting of either 4 items (for children aged 1–5 years, rated from 0-Never to 4-Always [35] or 5 items (for children aged 6–17 years, rated from 0-Not true to 3-definitively true [36]. *Attachment disordered behaviors* will be assessed using The Disturbances of Attachment Interview (DAI) [37], a semi-structured interview administered to the foster family. The DAI aims to gather information about the symptoms of Reactive Attachment Disorder and Disinhibited Social Engagement Disorder. It consists of 12 items, each coded on a scale of 0 (rarely or minimally), 1 (sometimes or somewhat), or 2 (clearly), based on the severity and frequency of behaviors described by the caregiver.

#### Child safety and permanency

Child safety and stability will be assessed by caseworkers using questionnaires specifically developed for this research project, drawing on previous work [38]. For *child safety*, which pertains to safeguarding children from harm, abuse, neglect, and various risks that may jeopardize their physical, emotional, or psychological well-being, caseworkers will evaluate both the physical and emotional environments within foster care over the preceding three months. The questionnaire comprises seven items rated on a 5-point scale, ranging from 1 (never) to 5 (always). Regarding *child stability*, which concerns the consistency and permanency experienced by a child in foster care, caseworkers will assess relational permanency. This involves evaluating emotional security (6 items), which pertains to the child’s opportunities to develop a sense of comfort and emotional support within the foster family, and claiming (4 items), which relates to the child’s opportunities to develop a sense of ownership and belonging within the family. Responses will be rated on a 5-point scale from 1 (totally disagree) to 5 (totally agree). Additionally, caseworkers will record incidents of placement breakdown (e.g., termination of foster care and reasons) and details about parental contacts (e.g., frequency of visits).

#### Foster family well-being and the experience of being a foster family

Caregivers will be asked to complete the Subjective Psychological Wellbeing–WHO-5 [39], which assesses *subjective psychological well-being* with 5 items, and the *Daily Hassles Questionnaire* [40], comprising 10 items to measure stress related to parenting activities. Additionally, *parental self-efficacy* and *satisfaction* concerning the foster care experience will be evaluated using the adapted version of the questionnaire Me as a Parent- Short Form (MaaP-SF) [41] and the Kansas Parental Satisfaction Scale (KPSS) [42]. The MaaP-SF consists of 4 items rated on a 5-point scale from 1 (totally disagree) to 5 (totally agree), while the KPSS comprises 3 items rated on a 7-point scale from 1 (extremely unsatisfied) to 7 (extremely satisfied). Furthermore, the intention to continue as a foster family will be assessed one month after the child has transitioned out of care.

#### Quality of relational care in the foster family

The quality of caregivers’ interactive behaviors with the child will be evaluated using two different assessment tools based on the child’s age. For children up to the age of 5, caregivers’ behaviors will be assessed using the well-known Ainsworth’s 9-point Insensitivity-Sensitivity and Cooperation-Intrusiveness subscales [43]. For school-aged children, the Dyadic Parent-Child Interaction Coding System [44] will be utilized. Caregivers will engage in structured play episodes with the child lasting 3–5 minutes each, well described in the literature [e.g., 44, 45], and designed to elicit various challenges for the dyad, such as free play, play with a challenging toy or clean up. Interactions will be recorded for subsequent coding of caregivers’ interactive behaviors. The "This is My Baby (Child) Interview" [46] is a semi-structured interview comprising nine questions aimed at evaluating the caregiver’s *acceptance* of the child, *commitment* to the child and to the caregiver-child interaction, and *awareness* of how the caregiver-child relationship impacts the child in both the present and the future. Caregivers will participate in these interviews, which will be audio-recorded for subsequent transcription and coding.

#### Sociodemographic information

Sociodemographic data of caseworkers (e.g., years of experience), children (e.g., age and sex; but also, e.g. exposure to early adversities, such as neglect and abuse) and their foster families (e.g., duration of fostering experience, number of children they are fostering) will be collected at the beginning of their participation in the study.

### Quality improvement evaluation

Implementation science underscores the significance of continuous quality improvement [8, 13], guided by data-driven strategies, in successfully implementing evidence-based interventions in child welfare. For instance, the Getting To Outcomes framework [8] advocates for a 10-step accountability approach to adopting a new child welfare practice model within a system. This model emphasizes that continuous quality improvement, alongside process and outcome evaluation, is essential for enhancing a practice model and strengthening services to better meet the needs of foster families and children. To facilitate professionals’ constructive participation in the continuous quality improvement process, strategies will be implemented. Specifically, four in-person sessions will be conducted with model providers between data collection time-points. These sessions will involve sharing and discussing the results obtained thus far, as well as collaboratively designing recommendations for improving the MIAF during its initial implementation phase.

### Data analysis plan

The project will gather a comprehensive dataset, requiring an analytical strategy that encompasses both qualitative and quantitative data analysis techniques. For qualitative data analysis, we will employ thematic analysis using a deductive-inductive approach, grounded in theoretical and empirical knowledge on the topic. We will follow recommended guidelines for coding, such as the Braun and Clarke step-based approach [47], and reporting, such as the Consolidated Criteria for Reporting Qualitative Research (COREQ) Checklist. Qualitative analysis will be facilitated using software such as NVivo and MAXQDA.

Regarding quantitative data, diverse analytical approaches will be employed. Descriptive analysis will examine demographic characteristics and study variables, presenting mean ± standard deviation (SD) for continuous variables and frequencies (n) and percentages for categorical variables. Correlational analysis will use Spearman and Pearson correlations, while the chi-square test will assess nominal variables. Growth Curve Modeling will examine the developmental trajectories of children in family foster care by analyzing their initial status and considering time-varying predictors (e.g., early adverse experiences, quality of care). Additionally, trajectories of family well-being will be examined. Moderation analyses will examine the influence of the MIAF (e.g., fidelity) on the relationship between predictors and outcome variables. Specifically, these analyses will explore the moderating effect of the MIAF model on the relationship between early child and family risk factors and subsequent outcomes. Statistical significance will be set at *p* < 0.05. Quantitative analysis will utilize software such as SPSS version 29.0 (SPSS Inc., Chicago, IL, USA) and R Statistics.

### Ethical considerations

Participation in the study will be voluntary and contingent upon obtaining written informed consent from all participants. Since children cannot provide consent themselves, their involvement will require consent from their legal guardians. Recognizing the vulnerability of children and the unique psychological and social contexts they may face, children’s verbal assent will also be sought after providing them with essential information. This will include the option to choose participation and assurance of the right to withdraw at any time. Trained researchers will closely monitor signs of distress from the child, and the session will only continue if the child is comfortable and engaged. The written consent form and initial briefing will provide legal representatives or adult participants with comprehensive information about the study’s objectives, methodology, potential risks and benefits, and their legal rights. Protection of personal data will be ensured by compliance with the EU General Data Protection Regulation. The University Institutional Review Board (06/2024) have approved the project.

### Data management

Data management protocols will ensure the security and integrity of all data collected. All data will be entered into secure databases with restricted access to authorized personnel only. To maintain data quality, range checks and double data entry processes will be implemented. Comprehensive data security measures will include regular backups, restricted access to stored data, and secure transfer methods to prevent unauthorized access or loss of data. These measures are designed to safeguard the confidentiality and integrity of the collected data throughout the duration of the project.

## Discussion

Family foster care in Portugal lags behind other Western European countries, remaining under-resourced and rarely implemented [4]. Urgent efforts are needed to shift towards family-based care, requiring new policies and resources to promote high-quality practices and ensure sustainable change [1, 2, 6]. Despite this imperative, Portugal lacks sufficient scientific evidence about family foster care and lacks a validated child welfare practice model to empower services and ensure better outcomes for children and families. This project aims to address these gaps by evaluating the initial implementation stage of the MIAF–i.e., a new practice model to promote high-quality family foster care in the country. By incorporating both outcome and process evaluation indicators, the project will provide evidence regarding the fidelity, feasibility, and acceptability of the MIAF. Additionally, it will identify barriers and facilitators of the MIAF’s implementation and assess the trajectories of both foster parents’ and children’s outcomes.

### Potential impact and innovation

Successful implementation of the MIAF has the potential to strengthen the virtually non-existent Portuguese family foster care system and support the delayed deinstitutionalization movement in the country. As an innovative child welfare practice model tailored specifically for family foster care, the MIAF comprehensively addresses the entire spectrum of care needs. The insights gained from this research initiative will serve as a foundation for the ongoing enhancement of MIAF. Consequently, this project has the capacity to advance evidence-based child welfare practices by refining processes and strategies to better serve vulnerable children and youth [9]. Also, by actively engaging stakeholders, including foster families, professionals, and children, the project ensures that their insights contribute to the model’s continuous improvement and inform implementation strategies [8].

Furthermore, the project’s comprehensive implementation and evaluation plan, integrating qualitative, quantitative, and longitudinal data, will significantly enhance our understanding of the strengths and weaknesses of the MIAF. This multifaceted approach will provide significant guidance for aligning the MIAF with Portugal’s broader child welfare system. With a multidisciplinary team leading the effort, the project is well-positioned to advance research in family foster care and implementation science, thereby informing public policies aimed at safeguarding the well-being of vulnerable children.

### Limitations and challenges

The protocol outlines a robust research design and implementation strategy, yet several limitations should be acknowledged. Firstly, the absence of a randomized controlled trial may pose challenges in establishing causal relationships between the MIAF model and observed outcomes. However, it is essential to emphasize that assessing the initial implementation of the MIAF will offer invaluable insights to enhance the practice model. This step is crucial before considering scaling up or conducting a randomized control trial, as strongly recommended by implementation science frameworks [8, 10]. Additionally, the study’s focus on specific geographic regions within Portugal may restrict the generalizability of findings to the entire country. However, it also allows for a deeper consideration of the unique characteristics and context-specific factors present in each region, which can provide valuable insights into the implementation and effectiveness of the MIAF in diverse settings. Moreover, the voluntary nature of participation may introduce selection bias, as participants who opt-in may differ systematically from those who decline. Furthermore, attrition over the study duration could impact the validity and reliability of longitudinal data analyses. Lastly, while the multidisciplinary team enriches the project with diverse expertise, it may also present challenges related to coordination and communication among team members with diverse backgrounds and expertise.

## References

[1] Desmond C, Watt K, Saha A, Huang J, Lu C. Prevalence and number of children living in institutional care: Global, regional, and country estimates. Lancet Child Adolesc Health. 2020;4:370–377. doi: 10.1016/S2352-4642(20)30022-5 32151317

[2] Van IJzendoorn MH, Bakermans-Kranenburg MJ, Duschinsky R, Fox NA, Goldman PS, Gunnar MR, et al. Institutionalisation and deinstitutionalisation of children 1: A systematic and integrative review of evidence regarding effects on development. Lancet Psychiatry. 2020;7:703–720. doi: 10.1016/S2215-0366(19)30399-2 32589867

[3] Zeanah CH, Humphreys KL, Fox NA, Nelson CA. Alternatives for abandoned children: Insights from the Bucharest Early Intervention Project. Curr Opin Psychol. 2017;15:182–188. doi: 10.1016/j.copsyc.2017.02.024 28813259 PMC5607636

[4] Instituto Segurança Social, Instituto Público. CASA 2023. Relatório de Caracterização Anual da Situação de Acolhimento das Crianças e Jovens. Lisboa, Portugal: ISSIP; 2022.

[5] United Nations General Assembly. United Nations Guidelines for the Alternative Care of Children. https://resourcecentre.savethechildren.net/pdf/5416.pdf/.

[6] Harlow E. Children’s rights, deinstitutionalisation and the development of foster care services across the world. Pract Soc Work Action. 2021.

[7] Anghel R, Herczog M, Dima G. The challenge of reforming child protection in Eastern Europe: The cases of Hungary and Romania. Psychosocial Interv. 2013;22:239–249.

[8] Barbee AP, Christensen DN, Antle B, Wandersman A, Cahn K. Successful adoption and implementation of a comprehensive casework practice model in a public child welfare agency: Application of the Getting to Outcomes (GTO) model. Child Youth Serv Rev. 2011;33:622–633.

[9] Pecora PJ, Kessler RC, Williams J, Downs AC, English DJ, White J, et al. What works in family foster care? Key components of success from the Northwest foster care alumni study. New York and Oxford, England: Oxford University Press; 2010.

[10] Sanclimenti JG, Caceda-Castro LE, DeSantis JP. Child welfare practice model implementation projects: Lessons learned. J Public Child Welfare. 2017;11:279–298.

[11] Antle BF, Barbee AP, Christensen DN, Martin MH. Solution-based casework in child welfare: Preliminary evaluation research. J Public Child Welfare. 2008;2:197–227.

[12] Antle BF, Christensen DN, van Zyl MA, Barbee AP. The impact of the Solution Based Casework (SBC) practice model on federal outcomes in public child welfare. Child Abuse Negl. 2012;36:342–353. doi: 10.1016/j.chiabu.2011.10.009 22502985

[13] Albers B, Mildon R, Lyon AR, Shlonsky A. Implementation frameworks in child, youth, and family services–Results from a scoping review. Child Youth Serv Rev. 2017;81:101–116.

[14] Rogers EM. Diffusion of innovations. 5th ed. New York: Free Press; 2003.

[15] Akin BA, Brook J, Lloyd MH, Bhattarai J, Johnson-Motoyama M, Moses M. A study in contrasts: Supports and barriers to successful implementation of two evidence-based parenting interventions in child welfare. Child Abuse Negl. 2016;57:30–40. doi: 10.1016/j.chiabu.2016.06.002 27288761

[16] Weeks A. Important factors for evidence-based implementation in child welfare settings: A systematic review. J Evid-Based Soc Work. 2021;18:129–154. doi: 10.1080/26408066.2020.1807433 32893742

[17] Negrão M, Moreira M, Veríssimo L, Veiga E. Conhecimentos e perceções públicas acerca do acolhimento familiar: Contributos para o desenvolvimento da medida. Análise Psicológica. 2019;37:81–92.

[18] Pinto VS, Luke N. The role of foster carers in England and Portugal: Is it solely a parenting role? Child Soc. 2022;36:249–265.

[19] Vanschoonlandt F, Vanderfaeillie J, Van Holen F, De Maeyer S, Robberechts M. Parenting stress and parenting behavior among foster mothers of foster children with externalizing problems. Child Youth Serv Rev. 2013;35:1742–1750.

[20] Gabler S, Kungl M, Bovenschen I, Lang K, Zimmermann J, Nowacki K, et al. Predictors of foster parents’ stress and associations to sensitivity in the first year after placement. Child Abuse Negl. 2018;79:325–338. doi: 10.1016/j.chiabu.2018.02.009 29510347

[21] Gabler S, Bovenschen I, Lang K, Zimmermann J, Nowacki K, Kliewer J, et al. Foster children’s attachment security and behavior problems in the first six months of placement: Associations with foster parents’ stress and sensitivity. Attach Hum Dev. 2014;16:479–498. doi: 10.1080/14616734.2014.911757 24785376

[22] Spangler G, Bovenschen I, Jorjadze N, Zimmermann J, Werner A, Riedel N, et al. Inhibited symptoms of Attachment Disorder in children from institutional and foster care samples. Attach Hum Dev. 2019;21:132–151. doi: 10.1080/14616734.2018.1499210 30033854

[23] Leathers SJ, Spielfogel JE, Geiger J, Barnett J, Vande Voort BL. Placement disruption in foster care: Children’s behavior, foster parent support, and parenting experiences. Child Abuse Negl. 2019;91:147–159. doi: 10.1016/j.chiabu.2019.03.012 30889437

[24] Pereira M, Negrão M, Soares I, Mesman J. Decreasing harsh discipline in mothers at risk for maltreatment: a randomized control trial. Infant Ment Health J. 2014;35:604–613. doi: 10.1002/imhj.21464 25798509

[25] Schofield G, Beek M. The secure base model: Promoting attachment and resilience in foster care and adoption. BAAF; 2014.

[26] Ball B, Sevillano L, Faulkner M, Belseth T. Agency, genuine support, and emotional connection: Experiences that promote relational permanency in foster care. Child Youth Serv Rev. 2021;121:105852.

[27] Proctor E, Silmere H, Raghavan R, Hovmand P, Aarons G, Bunger A, et al. Outcomes for implementation research: conceptual distinctions, measurement challenges, and research agenda. Adm Policy Ment Health. 2011;38(2):65–76. doi: 10.1007/s10488-010-0319-7 20957426 PMC3068522

[28] Mettert K, Lewis C, Dorsey C, Halko H, Weiner B. Measuring implementation outcomes: An updated systematic review of measures’ psychometric properties. Implementation research and practice. 2020;1:2633489520936644. doi: 10.1177/2633489520936644 37089128 PMC9924262

[29] Griffiths R. The abilities of young children: A comprehensive system or mental measurement for the first eight years of life. The Test Agency Limited; 1984.

[30] Wechsler D. Wechsler Preschool and Primary Scales of Intelligence. CEGOC; 2003.

[31] Sattler JM. Assessment of children: Revised and updated third edition. Jerome M. Sattler Publisher, Inc; 1992.

[32] Sheldrick RC, Henson BS, Neger EN, Merchant S, Murphy JM, Perrin EC. The baby pediatric symptom checklist: development and initial validation of a new social/emotional screening instrument for very young children. Acad Pediatr. 2013;13(1):72–80. doi: 10.1016/j.acap.2012.08.003 23092547 PMC3763819

[33] Sheldrick RC, Henson BS, Merchant S, Neger EN, Murphy JM, Perrin EC. The Preschool Pediatric Symptom Checklist (PPSC): development and initial validation of a new social/emotional screening instrument. Acad Pediatr. 2012;12(5):456–467. doi: 10.1016/j.acap.2012.06.008 22921494 PMC3907071

[34] Murphy JM, Bergmann P, Chiang C, Sturner R, Howard B, Abel MR, et al. The PSC-17: Subscale scores, reliability, and factor structure in a new national sample. Pediatrics. 2016;138(3):1–8. 10.1542/peds.2016-0038 PMC5005018 27519444

[35] Waters KA, Salinas-Miranda A, Kirby RS. The association between parent-child quality time and children’s flourishing level. J Pediatr Nurs. 2023;73:e187–e196. doi: 10.1016/j.pedn.2023.09.008 37775429

[36] Bethell CD, Gombojav N, Whitaker RC. Family resilience and connection promote flourishing among US children, even amid adversity. Health Aff. 2019;38(5):729–737. doi: 10.1377/hlthaff.2018.05425 31059374

[37] Zeanah C, Smyke A, Koga SF, Carlson E., The BEIP Core Group. Attachment in institutionalized and community children in Romania. Child Dev. 2005; 76(5):1015–1028. doi: 10.1111/j.1467-8624.2005.00894.x 16149999

[38] Mikolajczak M, Brianda ME, Avalosse H, Roskam I. Consequences of parental burnout: Its specific effect on child neglect and violence. Child Abuse Negl. 2018;80:134–145. doi: 10.1016/j.chiabu.2018.03.025 29604504

[39] Topp CW, Østergaard SD, Søndergaard S, Bech P. The WHO-5 Well-Being Index: a systematic review of the literature. Psychother Psychosom. 2015;84(3):167–176. doi: 10.1159/000376585 25831962

[40] Kanner A, Coyne J, Schaefer C, Lazarus R. Comparison of two models of stress measurement: daily hassles and uplifts *versus* major life events. J Behav Med. 1981; 4:1–39. 7288876 10.1007/BF00844845

[41] Matthews J, Millward C, Hayes L, Wade C. Development and Validation of a Short-Form Parenting Self-Efficacy Scale: Me as a Parent Scale (MaaPs-SF). J Child Fam Stud. 2022;1–11.

[42] Schumm W, Hall J. Kansas parental satisfaction scale (KPS). In: Fischer J, Corcoran KJ, eds. Measures for Clinical Practice and research: A sourcebook. 4th ed. NY. Oxford University Pr; 2007. p. 357–358.

[43] Ainsworth MDS, Blehar MC, Waters E, Wall S. Patterns of attachment: A psychological study of the strange situation. Hillsdale, NJ: Lawrence Erlbaum Associates; 1978.

[44] Nelson MM, Olsen B. Dyadic Parent–Child Interaction Coding System (DPICS): An adaptable measure of parent and child behavior during dyadic interactions. In: Niec L, ed. Handbook of Parent-Child Interaction Therapy. Springer; 2018. p. 285–302.

[45] Baptista J, Silva JR, Marques S, Martins C, Soares I. Early maltreatment and current quality of relational care predict socioemotional problems among institutionalized infants and toddlers. Infant Ment Health J. 2018;39(6):718–729. doi: 10.1002/imhj.21741 30339735

[46] Dozier M, Stoval KC, Albus KE, Bates B. Attachment for infants in foster care: The role of caregiver state of mind. Child Dev. 2001;72:1467–1477. doi: 10.1111/1467-8624.00360 11699682

[47] Braun V, Clarke V. Conceptual and design thinking for thematic analysis. Qual Psychol. 2022;9(1):3–26.

